# P-711. Testing for SARS-CoV-2, Respiratory Syncytial Virus and Human Metapneumovirus among patients hospitalized with acute respiratory tract infection — United States, 2022–2025

**DOI:** 10.1093/ofid/ofaf695.923

**Published:** 2026-01-11

**Authors:** Ian D Plumb, Pauline Terebuh, Quangqiu Wang, Regina Simeone, Jefferson M Jones, Rebecca J Free, Amanda B Payne, Yasir Tarabichi, Fiona P Havers, David C Kaelber

**Affiliations:** Division of Foodborne, Waterborne, and Environmental Diseases, Centers for Disease Control and Prevention, Atlanta, GA, Atlanta, Georgia; Case Western Reserve University School of Medicine, Cleveland, Ohio; Case Western Reserve University, Cleveland, Ohio; Centers for Disease Control and Prevention, Atlanta, Georgia; CDC, Atlanta, Georgia; Centers for Disease Control and Prevention, Atlanta, Georgia; CDC, Atlanta, Georgia; MetroHealth Systems, Cleveland, Ohio; Centers for Disease Control and Prevention, Atlanta, Georgia; MetroHealth Medical Center/ Case Western Reserve University, Cleveland, Ohio

## Abstract

**Background:**

Understanding clinical testing for SARS-CoV-2, respiratory syncytial virus (RSV) and human metapneumovirus (hMPV) can guide use of healthcare data for public health surveillance. We described testing patterns of these viruses over time by U.S. region among patients hospitalized with acute respiratory tract infection (ARI).

**Figure:**
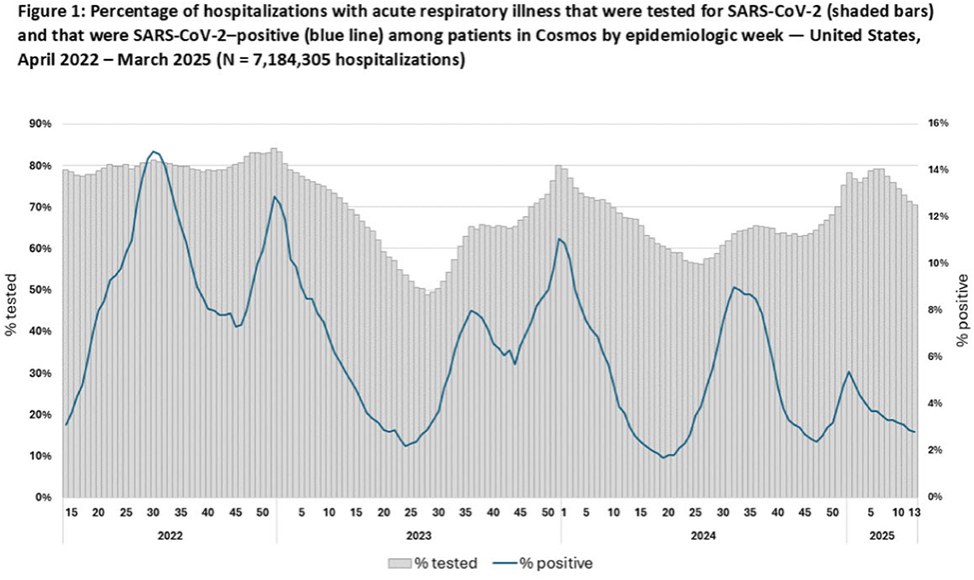

**Figure:**
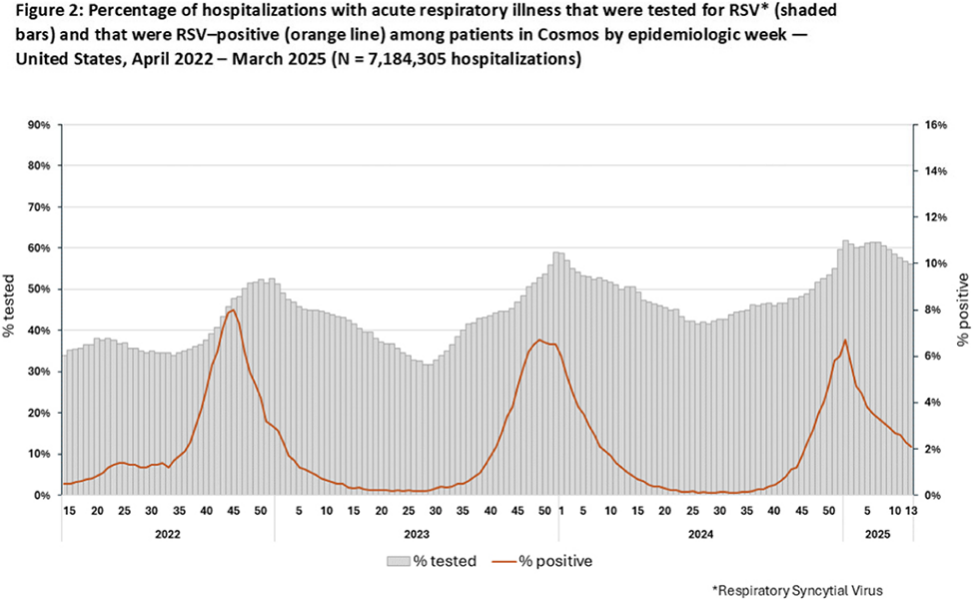

**Methods:**

We performed descriptive analyses of data from Cosmos, a de-identified electronic health records platform representing >298 million patients in the United States. We described virologic testing for SARS-CoV-2, RSV and hMPV (using LOINC codes) from 10 days before to 3 days after hospitalization with ARI (identified using ICD-10 codes) during April 3, 2022–March 29, 2025. For each epidemiologic week, we reported percentages of hospitalizations tested and test positivity if tested. We reported overall testing during the analysis period by age group, sex, race and ethnicity, social vulnerability index quartile, and Health and Human Services (HHS) region of residence.

**Figure:**
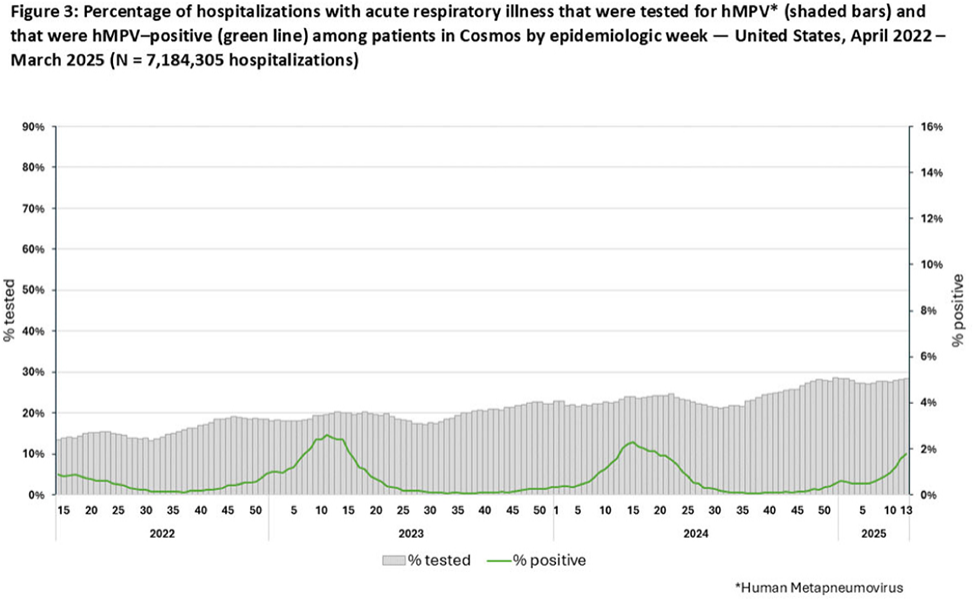

**Figure:**
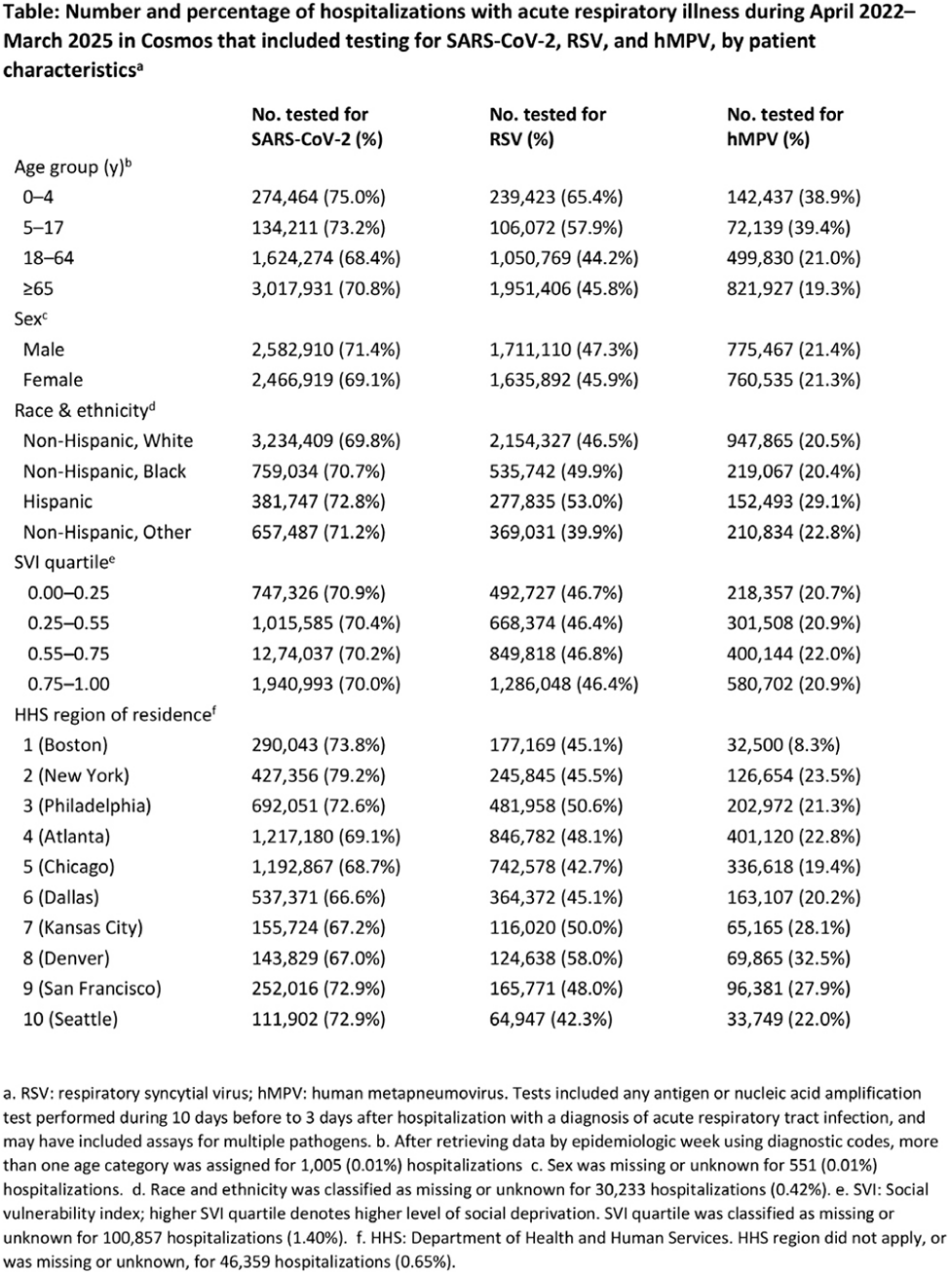

**Results:**

During 2022–2025, 4,782,624 patients had a total of 7,184,305 hospitalizations with ARI. From year 1 (April 3, 2022–March 26, 2023) to year 3 (March 31, 2024–March 29, 2025), percentages of hospitalizations tested decreased from 79% to 67% for SARS-CoV-2 but increased from 42% to 51% for RSV and from 17% to 25% for hMPV. For SARS-CoV-2 and RSV, peaks in positivity generally corresponded to periods of increased testing; peaks in hMPV positivity corresponded less clearly to testing levels (Figures 1–3). Testing varied across age groups by 4% for SARS-CoV-2, 21% for RSV, and 20% for hMPV; testing for RSV and hMPV was higher among children than adults. Percentages of hospitalizations with testing varied across HHS regions by 12% for SARS-CoV-2, 16% for RSV and 24% for hMPV (Table). Testing by other characteristics is summarized in the Table.

**Conclusion:**

Testing for SARS-CoV-2 declined during 2022-2025 but remained higher than testing for RSV and hMPV, which both increased over the same period. Testing varied by positivity and region, and was higher among children than adults for RSV and hMPV. These findings can inform use of healthcare data to estimate illness burdens from SARS-CoV-2, RSV, and hMPV. Awareness of seasonal variation in test positivity might help inform clinical testing.

**Disclosures:**

All Authors: No reported disclosures

